# COVID-19 Diagnosis from Chest X-ray Images Using a Robust Multi-Resolution Analysis Siamese Neural Network with Super-Resolution Convolutional Neural Network

**DOI:** 10.3390/diagnostics12030741

**Published:** 2022-03-18

**Authors:** Happy Nkanta Monday, Jianping Li, Grace Ugochi Nneji, Saifun Nahar, Md Altab Hossin, Jehoiada Jackson, Chukwuebuka Joseph Ejiyi

**Affiliations:** 1 School of Computer Science and Engineering, University of Electronic Science and Technology of China, Chengdu 611731, China; mh.nkanta@std.uestc.edu.cn; 2 School of Information and Software Engineering, University of Electronic Science and Technology of China, Chengdu 611731, China; ugochinneji@std.uestc.edu.cn (G.U.N.); kofijackson@uestc.edu.cn (J.J.); cjejiyi@std.uestc.edu.cn (C.J.E.); 3 Department of Information System and Technology, University of Missouri-St. Louis, St. Louis, MO 63121, USA; snnnm@umsl.edu; 4 School of Management and Economics, University of Electronic Science and Technology of China, Chengdu 611731, China; altabbd@uestc.edu.cn

**Keywords:** chest X-ray (CXR), COVID-19, convolutional neural network, multi-resolution analysis, super resolution, Siamese network

## Abstract

Chest X-ray (CXR) is becoming a useful method in the evaluation of coronavirus disease 19 (COVID-19). Despite the global spread of COVID-19, utilizing a computer-aided diagnosis approach for COVID-19 classification based on CXR images could significantly reduce the clinician burden. There is no doubt that low resolution, noise and irrelevant annotations in chest X-ray images are a major constraint to the performance of AI-based COVID-19 diagnosis. While a few studies have made huge progress, they underestimate these bottlenecks. In this study, we propose a super-resolution-based Siamese wavelet multi-resolution convolutional neural network called COVID-SRWCNN for COVID-19 classification using chest X-ray images. Concretely, we first reconstruct high-resolution (HR) counterparts from low-resolution (LR) CXR images in order to enhance the quality of the dataset for improved performance of our model by proposing a novel enhanced fast super-resolution convolutional neural network (EFSRCNN) to capture texture details in each given chest X-ray image. Exploiting a mutual learning approach, the HR images are passed to the proposed Siamese wavelet multi-resolution convolutional neural network to learn the high-level features for COVID-19 classification. We validate the proposed COVID-SRWCNN model on public-source datasets, achieving accuracy of 98.98%. Our screening technique achieves 98.96% AUC, 99.78% sensitivity, 98.53% precision, and 98.86% specificity. Owing to the fact that COVID-19 chest X-ray datasets are low in quality, experimental results show that our proposed algorithm obtains up-to-date performance that is useful for COVID-19 screening.

## 1. Introduction

The coronavirus disease 2019 (COVID-19) epidemic resulted from a novel strain of coronavirus that had not been previously diagnosed in humans and was first discovered in late December 2019; since then, it has spread rapidly, infecting over 410 million individuals globally, killing over 5.8 million people as of 13 February 2022 [1,2]. The gold standard for identifying COVID-19 is now Reverse Transcriptase Quantitative Polymerase Chain Reaction (RTq-PCR) tests [3,4]. Small quantities of viral RNA are collected from a nasal twirling, increased in size, and measured during this test, with virus confirmation displayed visually with a fluorescent dye. Unfortunately, the RTq-PCR test is time-consuming and procedural, requiring roughly two days for completion. Some researchers have also reported false-positive RTq-PCR [5]. Other testing methods include vision-based technology such as computed tomography (CT) imaging [6] and CXR imaging [7,8]. In a clinical review of COVID-19, CT and CXR scans have shown to be successful [9,10,11,12,13,14]. However, COVID-19 detection based on CT scan is time-consuming and requires experts’ involvement. CT scanning equipment is often troublesome to operate for COVID-19 patients since they must often be moved to the CT room. The machines must be thoroughly cleaned after each use, and there is a higher risk of radiation exposure [15].

CT has been successfully used as a supportive method for COVID-19 condition evaluation, despite the fact that it is not approved as a basic diagnostic means [6]. Ground-glass opacities (GGO) at the beginning stage and developing stage, air space merging during the apex stage, bronchovesicular thickening in the wound, and stretching bronchiectasis evident during the intake stage are all common CT findings [15]. Chest X-ray (CXR) imaging, on the other hand, is relatively inexpensive and widely used for lung infection diagnosis as well as for COVID-19 detection [16]. Owing to the rapid growth of COVID-19 patients, physicians and radiologists are in short supply. To this end, developing artificial intelligence techniques for computer-assisted COVID-19 classification with CXR images is a top priority. Along with ample data, convolutional neural networks (CNNs) have achieved up-to-date results in the fields of biomedical engineering and healthcare [3,17,18]. This level of efficiency is achieved by practising on labeled data and fine-tuning the millions of parameters that make up the system. Because of the large number of parameters, CNNs can easily overfit on small amounts of data.

As a consequence, generalization efficiency is reciprocal to the size of the labeled data. Tiny datasets are the most challenging task in the healthcare imaging domain because of the restricted quantity and variety of samples [5,6,7]. Medical data mining is a time-consuming and costly procedure that necessitates the involvement of radiologists and researchers [6]. Furthermore, especially with the present existence of the COVID-19 outbreak, adequate data of CXR images are troublesome to come by. However, in AI-based COVID-19 screening systems from chest X-ray imaging, there are two major problems; (1) low resolution (LR) is still a major challenge and (2) image quality is still a major concern as this may vary among samples, which often include noise and irrelevant annotations. The consequent of this is that the AI-based system will learn inconsistent and noisy information from the data, thereby missing the distinct features that would have been extracted for optimal classification.

In order to mitigate these setbacks, we propose a super-resolution convolutional neural network-based multi-resolution CNN Siamese framework for COVID-19 classification. The contributions of this work include: 1. To enhance the feature extraction robustness of the network, we propose an enhanced fast super-resolution convolutional neural network (EFSRCNN) to recapture high-resolution (HR) counterparts of low-resolution (LR) images, from which it is able to extract distinct details. The EFSRCNN handles the problem of low-quality images and helps to generate super-resolution images as well as improving the PSNR. 2. To our knowledge, this paper is the first pioneering work that introduces an end-to-end super-resolution CNN with a Siamese-based multi-resolution neural network framework as a mutual learning approach for COVID-19 classification from CXR images. The proposed architecture achieves much higher diagnosis accuracy. The subsequent structure of this paper is coordinated as follows: in Section 2, we analyze related essays. We then give a detailed description of the materials and methods in Section 3. Section 4 contains details of the experimental setup, and implementation information. We conduct robustness evaluation in Section 5. The findings as well as other relevant discussion are presented in Section 6. Section 7 brings this paper to an end.

## 2. Related Works

In the past, artificial intelligence-based approaches have been utilized to reliably diagnose a range of ailments from healthcare images, surpassing human-centered diagnosis in many instances. Deep learning has increasingly been used to detect COVID-19 in medical imaging. A deep neural ensemble learning network with random forest called EDRnet was suggested in [19] to predict COVID-19 from samples of routine blood. The authors chose 28 blood biomarkers and utilized common attributes such as the age and gender of the patients as the input data for the model. The authors claimed that their model achieved 100% sensitivity, 91% specificity, and 92% accuracy. The authors in [20] suggested different classification models to prioritize symptomatic patients for COVID-19 early detection using metadata such as gender and fever as input data for the models. An average of 90% accuracy was obtained. A machine learning model was proposed in [21] to differentiate between confirmed patients with severe and non-severe COVID-19 infection utilizing multiple clinical features. Prediction accuracy of 96% was reported when the authors adopted a random forest model with the most important multi-modal attribute features, such as age, hypertension, cardiovascular disease, gender, diabetes, and lactate dehydrogenase.

Quite an interesting work was presented by the authors in [22] to compare and quantify people’s preferences for AI clinicians and traditional clinicians. The authors adopted a method of propensity score matching to match similar demographic characteristics of two different categories of respondents. The authors reported that the AI diagnosis technique outweighed human clinician diagnosis, with 95% of the respondents believing that the AI-based clinician method achieves better accuracy at low expense. A hybrid deep learning and machine learning model using a multi-modal fusion approach with three machine learning classifiers was constructed in [23] to extract 10 high-level representation features from CT exams combined with low-dimensional medical and lab testing data to distinguish between COVID-19 and other forms of viral pneumonia as well as healthy patients. The overall prediction accuracy ranged from 95 to 97%. The study in [24] suggested a machine learning approach to rule out routine blood tests as the only data for COVID-19 diagnosis among adults in emergency units. The authors claimed that their method achieved 98% sensitivity and 97% specificity by integrating multi-center medical data collected from the emergency unit’s laboratory. A deep transfer learning network with different pre-trained models as the backbone, called FCONet, was proposed in [25] to classify COVID-19 and other pneumonia diseases using CT images. The pre-trained network of FCONet with ResNet50 as a backbone obtained 99% accuracy.

A concise review on the effectiveness of the AI-based diagnosis of COVID-19 is presented in [26]. The authors emphasized the importance of timely and early prognosis and diagnosis of COVID-19 patients to curb the spread of the virus and thus reduce the burden on the healthcare system and clinicians. The authors reported that deep learning models have achieved high sensitivity results compared to human clinicians diagnosis. An online AI-based approach of statistical deep learning techniques to predict COVID-19 was developed in [27] using two publicly available datasets. A multi-level pipeline model based on a deep neural network approach was presented in [28] to classify COVID-19 and other forms of pneumonia using chest X-ray images. The authors of this study adopted the ResNet50 pre-trained model as the backbone network. The authors reported training and test accuracy of 96% and 92%, respectively. A residual CNN architecture was proposed to classify COVID-19 from non-COVID-19. The model was trained on data from two publicly accessible sources [29,30,31]. The model achieved 80% sensitivity and 94.9% specificity, with only 10 instances of COVID-19 images. To minimize the number of false negatives, future models should increase the sensitivity according to this research. A modified Bayesian ResNet50 [30] architecture with weight descent was proposed in [31] to classify four categories of data into COVID-19, non-COVID-19, healthy, and pneumonia using 14 COVID-19 instances, in which two of the COVID-19 instances were incorrectly categorized in BCNNs and CNNs when dropping the weights at different points. Their best model for COVID-19 diagnosis had 86% sensitivity and 99% precision. The study pointed out that model efficiency can be enhanced by estimating uncertainty within predictions.

Three separate deep transfer learning networks were suggested in [32] to diagnose COVID-19 from among healthy cases using 50 instances each. In [33], the authors reported that Inception V3 achieved perfect results, as well as ResNet50 in [34], whereas in [35], Inception-ResNet V2 incorrectly classified one healthy instance as COVID-19 in a testing set of ten images from each class. The authors suggested the merits of fine-tuning deep learning models for COVID-19 investigation. An ImageNet [36] pre-trained 18-layer residual CNN was suggested in [37]. For classification, the CNN was accompanied by completely linked layers and sigmoid activation. At the end of the CNN, a separate anomaly detection mechanism was introduced. A cross-validation approach of two-fold threshold was adopted to record the specificity and sensitivity on 100 instances. The model achieved 72% sensitivity at 98% specificity and 96% sensitivity at 70% specificity. In conclusion, these frameworks tend to work admirably; indeed, due to the possibility of missing a COVID-19 diagnosis, increasing the model sensitivity is a top priority. Quite a number of models have been created to diagnose COVID-19 using CT and CXR. A COVID-19 contusion identification approach for the diagnosis of COVID-19 was suggested in [38], where the algorithm [39] was trained on professionally interpreted CT slices in order to extract COVID-19-infected areas using 11 suspected COVID-19 and 16 pneumonia patients. The authors claimed that their model performed similarly to a professional radiologist, with 99% sensitivity on 300 COVID-19 instances. A similar segmentation network was proposed in [40] to segment COVID-19 with accuracy of 91%. The authors suggested that their approach could be used to monitor the disease’s progression. COVID-19 was segmented and quantified using a combination of commercial software and deep learning in [41], with 96% AUC. A shared weighted ResNet50 model was proposed in [42] for each slice in a CT image. The max pooling layer combined the slices to create a feature vector for classification using 68 COVID-19 and 285 healthy instances, with 96% AUC.

A fine-tuned siamese network with modified enhanced super resolution GAN plus based on low quality chest X-ray images was suggested in [43] to identify COVID-19 instances from non-COVID-19, achieving 98.8%, precision of 98.6%, sensitivity of 97.5%, specificity of 98.9%, an F1 score of 97.8% and ROC AUC of 98.8% for the multi-class task, while for the binary class, the model achieves accuracy of 99.7%, precision of 98.9%, sensitivity of 98.7%, specificity of 99.3%, F1 score of 98.2% and ROC AUC of 99.7%.. According to [44], segmented scans were used to remove infection and lung fields, and the images were categorized according to infection size using a random forest infection size classifier on a five-fold cross-validation. The method achieved 94% AUC using 1657 COVID-19 and 1028 healthy patients. A 3D neural network was proposed in [45] to segment lesions before using a 2D ResNet network to classify them as COVID-19 or not. This approach was examined on datasets from two hospitals, achieving 99% AUC on 128 healthy and 154 COVID-19 exams. For CT slice classification, a ResNet152 integrated segmentation network was proposed in [46] to concentrate on the diseased area. This network achieved 98% AUC on local and public datasets with 1,071 healthy and 183 COVID-19 instances. A deep learning model was suggested in [47] for the segmentation of infection spots. These infected patch areas were fed as input to the ResNet18 network for classification using 60 instances of pneumonia and 30 COVID-19 instances. The model achieved 86% accuracy.

An inception network was suggested in [48] to diagnose COVID-19 using a private dataset of 100 instances each for healthy and pneumonia cases, whereas COVID-19 had only 10 instances, with 89% accuracy in the internal validation, while the external validation achieved accuracy of 83%. An attention module-based function pyramid network with ResNet50 was proposed in [49] using a private dataset of 27 COVID-19 and 24 healthy instances. The authors claimed that the model achieved 99% AUC and 93% sensitivity. However, the procedure achieved 95% AUC and 96% sensitivity on a dataset of 27 COVID-19 and 30 bacterial pneumonia instances. An interesting procedure suggested a deep learning model with a random forest classifier focused on measurable features to determine the magnitude of COVID-19 [50]. The procedure achieved overall accuracy of 87% using three-fold cross-validation on 176 instances.

A weakly supervised approach was proposed in [51] in which segmentation masks were produced automatically and, hence, the mask and CT image were passed into a 3D CNN for classification. The authors recorded 95% AUC using this procedure. In summary, most studies, including those using CXR imaging, rely on quite a few COVID-19 images from various sources, with no standardized protocols. The reason that AI innovation and clinical utility are minimal is that these studies apply previously established AI-based algorithms to new problems. In general, COVID-19 screening based on CT or CXT images has achieved a significant improvement according to [52]. Moreover, a number of models have utilized very few images—as low as 10 COVID-19 instances in the test set—while some used external validation owing to data scarcity. Building a system that can achieve better performance using fewer image data is necessary because it will permit the greater inclusion of uncommon data classes in the test dataset. The goal of this research is to build an AI-based model that is robust enough to utilize few and low-quality image instances and still achieve high performance.

## 3. Materials and Methods

### 3.1. Problem Statement

Thorough COVID-19 screening is essential in light of the imminent pandemic threat. A serious problem is encountered with regard to the insufficiency of COVID-19 test kits in many developed/rural locations, as well as the time it takes to produce the sample (correct) findings, which also in turn affects developing countries with under-equipped hospitals and clinics. Developing countries commonly lack sufficient COVID-19 kits, restricting primary healthcare clinics’ capacity to obtain, ship, and evaluate test findings, causing them to be dependent on more specialized institutes. To respond to the third wave of the pandemic, an automated and efficient supplemental technique is necessary to address the increasing demand for additional test cases in places with minimal access to antibody tests.

Many studies have shown that CT scans can detect ground-glass opacities and other chest characteristics that are more detailed than a normal chest X-ray. CT scans are not reliable for COVID-19 purposes due to infection management concerns associated with transferring patients to CT units, comparably high expenses (high purchase cost, installation, and repair of CT equipment), and poor system availability in rural locations. A chest X-ray (CXR) may, on the other hand, be utilized to detect COVID-19 [10] or other pneumonia outbreaks, as CXR imaging equipment is commonly available in emergency rooms, public health centers, and even rural clinics. Nonetheless, with AI-based CXR detection systems, there are two major bottlenecks. 1. The low-resolution (LR) features are an issue; 2. The acquired dataset samples usually consist of unnecessary details and blurry features.

Even experienced radiologists have difficulty distinguishing between the features of COVID-19 pneumonia and community-acquired bacterial pneumonia when reviewing chest X-ray images [10]. Furthermore, the influx of patients into hospital ERs during the pandemic, manual inspection of radiograph data, and accurate decision-making will all contribute to a difficult trade-off between accuracy and detection time, potentially exhausting the radiology unit and, as a matter of urgency, necessitating the use of an automated identification method. A third wave of COVID-19 activity would call for an increase in compact chest X-ray devices, as their widespread use would render CTs obsolete. We discuss the concerns raised previously and proposed a deep learning-based Siamese discrete wavelet multi-resolution with enhanced fast super-resolution convolutional neural network solution to address the third-wave challenges.

### 3.2. Datasets

**Figure 1:**
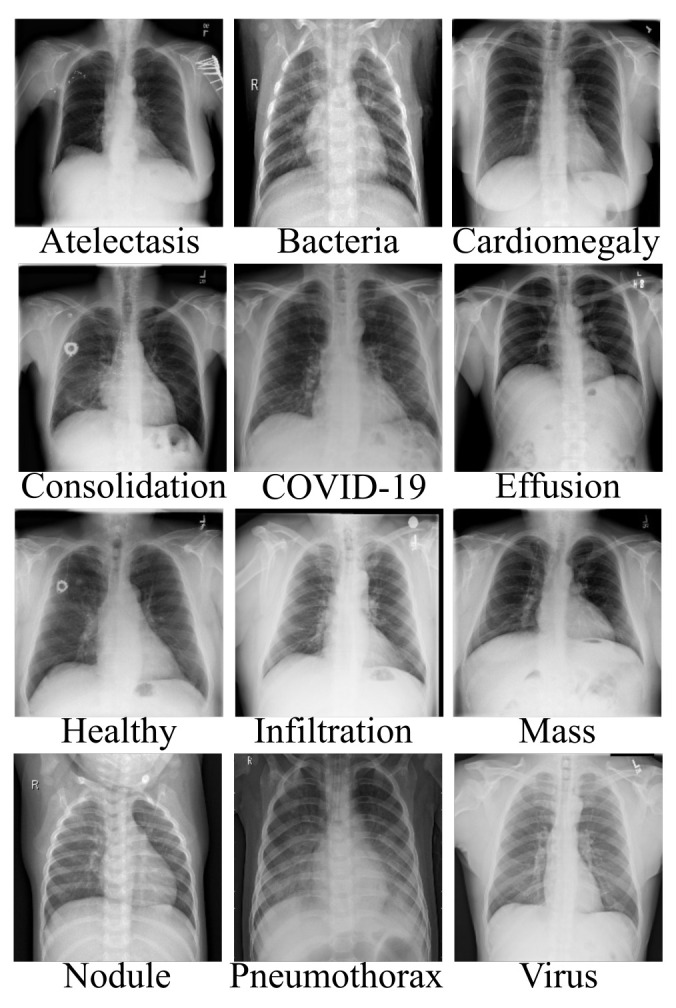
Data collection of chest X-ray images of different pneumonia-related illnesses including COVID-19.

Artificial intelligence (AI) has achieved a remarkable reputation in the field of clinical research. In the face of the current pandemic, artificial intelligence can assist healthcare workers in the process of disease detection, boosting the accuracy of identification methods at a fast rate and perhaps saving lives. The scarcity of appropriate data is perhaps the most significant barrier facing AI-based approaches. Since AI-based approaches are data-driven, a large amount of data is needed. The process of data collection is quite tedious as there are many ethics concerns from experts. Bearing this view in mind, we resorted to well-known and validated dataset repositories for the collection and compilation of the dataset. In this study, we collected chest X-ray data of different pneumonia-related illnesses from three different open sources [53,54,55]. As illustrated in Table 1, we collected 3616 scans of COVID-19 CXR from the COVID-19 radiography database [53]. In addition, we collected 3029 scans of bacterial pneumonia, 8851 scans of healthy patients, and 2983 scans of viral pneumonia from the Kaggle database of the Radiological Society of North America (RSNA) [54]. Moreover, we collected 74,999 scans of other pneumonia-related illnesses from the National Institute of Health (NIH) [55], as illustrated in Table 1, for the purpose of validating our proposed architecture for multiple classification problems. As indicated, there are approximately 90,983 CXR scans including COVID-19 and 10 other pneumonia-related illnesses as well as healthy instances. Since the number of each category of data class varies, as a result, we selected 1000 scans of CXR from each category, which sum up to 12,000 CXR images. Moreover, since the amount of CXR associated with each class is balanced, the dataset is partitioned into three sets of 60%, 20%, and 20% for training, validation, and testing, respectively. Figure 1 gives a visual representation of the dataset distribution.

### 3.3. COVID-19 Classification Architecture

The overall illustration of our proposed architecture, called COVID-SRWCNN, consists of two distinct stages. In the first stage, the enhanced fast super-resolution convolutional neural network (EFSRCNN) is utilized to reconstruct high-resolution CXR images from the low-resolution original CXR images. Secondly, the high-resolution CXR images are then passed as inputs to our proposed Siamese wavelet multi-resolution convolutional neural network (SWMRCNN) to extract and learn discriminative features for the diagnosis of COVID-19.

### 3.4. Enhanced Fast Super-Resolution Convolutional Neural Network (EFSRCNN)

**Figure 2:**
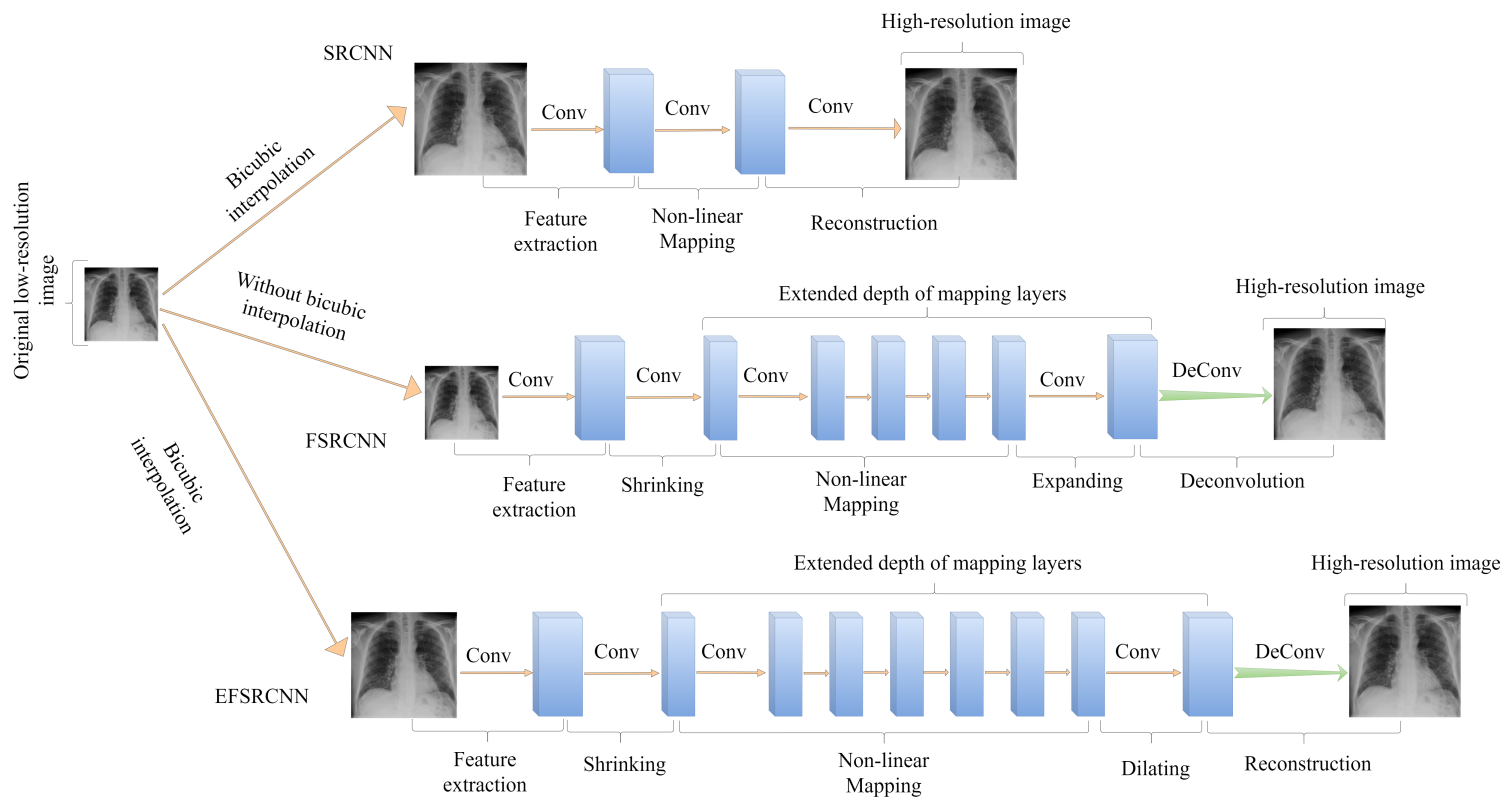
This figure shows the network architecture of the SRCNN, FSRCNN, and our proposed enhanced super-resolution framework called EFSRCNN. In a logical sense, our proposed model is centered on the merits of both the SRCNN and FSRCNN. First, EFSRCNN uses the bicubic interpolated version of the ground-truth low-resolution image as an input, similar to the process in SRCNN but different from the process in FSRCNN. Similar to FSRCNN, a deconvolutional layer is added at the end of the network to achieve up-sampling. Shrinking, mapping, and dilation phases of EFSRCNN replace the non-linear mapping phase in SRCNN and it is quite similar to the phases in FSRCNN. Nevertheless, EFSRCNN has a deeper network topology compared to FSRCNN. The sizes of the filters within the mapping layers are kept similar to FSRCNN. These enhancements give EFSRCNN higher performance while lowering the computational cost compared to SRCNN and FSRCNN.

The general procedure of SRCNN aims at extracting patches from the input in the first layer represented as high-dimensional feature vectors. The middle layer maps the feature vectors non-linearly to high-dimensional feature vectors and, thereafter, the final reconstruction layer then combines these features to create the final output image. Since the middle layer contributes the most to the network parameters, the size of the generated high-resolution image is directly proportional to the network complexity. Our proposed EFSRCNN is broken into five sections, with which the first four sections are convolutional layers, followed by a deconvolutional layer, which is the fifth section, as indicated in Figure 2, which includes feature extraction, shrinking, mapping, dilation, and deconvolution.

#### 3.4.1. Feature Extraction

This section is comparable to the first portion of SRCNN. but different from FSRCNN. EFSRCNN extracts features from the original LR image after interpolating them. The small LR input is denoted as $Y_{z}$ to distinguish it from SRCNN. Each patch of the input is represented as a high-dimensional feature vector using convolution with the first set of filters. On the choice of selecting parameters such as filter size $f_{k}$ and the number of channels $c_{k}$, we refer to SRCNN. Without much information loss, we adopted a filter size of 3. In SRCNN, the first layer’s filter size is set at 9. It is worth noting that these filters are applied to the upscaled image *Y*. Because the majority of the pixels in *Y* are interpolated from $Y_{z}$, a 50% patch in $Y_{z}$ might encompass nearly all of the information in a 99% patch in *Y*. We use SRCNN to set the number of channels $c_{k}$ for the first layer to be 1.

#### 3.4.2. Shrinking

In SRCNN, the feature extraction stage is usually followed by the mapping step, after which the high-dimensional LR features are directly mapped to the HR feature space. Nevertheless, because the LR feature dimension is normally quite large, the mapping step’s computational complexity is quite high. Similar to FSRCNN, we introduce the 1×1 convolutional layer after the feature extraction layer, called the shrinking layer, to shrink the interpolated LR feature dimension with a filter size of 1, which acts as a linear combination within the interpolated LR features.

#### 3.4.3. Non-Linear Mapping

The non-linear mapping step is the most critical aspect that influences SR performance. The number of filters in a layer (width) and the number of layers (depth) of the mapping layer are the most influential parameters. To achieve high performance comparable to SRCNN and FSRCNN, we increase the depth of the mapping layer to 6 convolutional layers with a 3×3 filter size each to maintain consistency.

#### 3.4.4. Dilating

In contrast to the shrinking layer, the dilating layer acts in the reverse direction. For the sake of computing performance, the shrinking procedure reduces the number of the interpolated LR feature dimensions. The ultimate restoration quality will be poor if we generate the HR image directly from these low-dimensional characteristics. As a result, after the mapping section, we add a dilating layer to broaden the HR feature dimension. To keep the shrinking layer consistent, we use 1×1 filters, the same number as the interpolated LR feature extraction layer.

#### 3.4.5. Reconstruction

The final layer is a deconvolution layer, which uses a collection of deconvolution filters to up-sample and aggregate the prior features. The deconvolution can be thought of as the reverse process of the convolution. The filter is convolved with the image using a stride of 2 for convolution, and the output is $\frac{1}{2}$ times the input. In contrast, if we swap the input and output positions, the result will be 2 times the input. Surprisingly, the inverted network functions similarly to a down-scaling operator that accepts the HR image and produces an LR image. The deconvolution layer is then transformed into a convolution layer with a stride of 2. We use 9×9 filters in order to maintain consistency with the first layer of SRCNN because it collects features from the HR images. Similarly, the deconvolution filters have a spatial size of 9 when we reverse the process.

#### 3.4.6. Cost Function

In consideration of the network parameters, the mean squared error is used to minimize the loss between the recaptured high-resolution images F(Y;Ψ) and the actual images *X* for a given range of high-resolution images $X_{k}$ and their counterpart low-resolution images $Y_{k}$; the mean squared error loss function is given in Equation (1).

(1) $$L\left(\Psi \right)=\frac{1}{n}\sum_{k=1}^{n}\;||F\left(Y_{k};\Psi \right)-X_{k}{\left|\right|}^{2}$$

The training samples in the set are denoted by *n*. A high PSNR is achieved in this case by utilizing the MSE loss function, which is a well-known metric for assessing image restoration efficiency.

#### 3.4.7. Differences with Other Super-Resolution Methods

It is worth illustrating how the merits of both SRCNN and FSRCNN translated to EFSRCNN within a few steps. We present the network topology of SRCNN and FSRCNN. We also illustrate the performance of these networks in terms of PSNR trained on a chest X-ray dataset. First, we maintain the same pre-processing operation as SRCNN in the first layer. Secondly, we increase the depth of the mapping layers as compared to FSRCNN by adding 2 more convolutional layers, resulting in a total of 6 mapping layers. It is well known that the depth of the layers affects the performance of the network. We adopt 6 thin layers, thus obtaining satisfactory results of 33.24 dB with fewer parameters. Finally, we utilize small filter sizes and fewer filters to achieve a speed of 52.1×. Our proposed enhanced fast super-resolution network outperforms SRCNN and FSRCNN by a large margin. The high performance of our method is attributed to the number of filter sizes, as presented in Table 2.

### 3.5. Siamese Wavelet Multi-Resolution Convolutional Neural Network (SWMRCNN)

**Figure 3:**
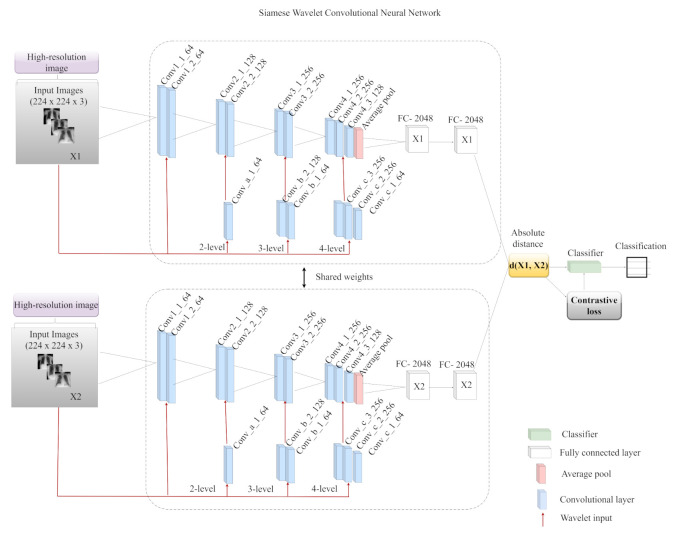
Our proposed Siamese wavelet multi-resolution convolutional neural network.

Using two similar multi-resolution wavelet convolutional neural networks with the same weights, our proposed COVID-19 classification network learns fixed-length representations. To minimize the computational cost and model complexity, we built each identical CNN from scratch in our experiment, as shown in Figure 3. The architecture consists of two parts; the first part is the wavelet decomposition multi-resolution analysis for image pre-processing and filtering, while the second part is the convolutional neural network for feature learning and classification. The first part tries to capture detailed features of the image and eliminate the noisy content present in the image by means of a filtering technique. These high- and low-pass filters generate the detail and approximate components from the original image with the help of the wavelet and scaling function by down-sampling with a scale factor of 2. The generated detail component is now the new input image fed to the convolutional neural network for feature learning and classification.

The generated approximate component is passed to the second-level decomposition stage, where it is further decomposed to generate second-level detail and approximate components. This process is repeated for four levels. The second part is subdivided into two pathways: the feature learning block and the concatenation block. The feature learning block consists of 9 convolutional layers, where each convolutional layer is followed by batch normalization and a ReLU activation function. We did not utilize max pooling in our model; rather, we added global average pooling after the last convolutional layers and a dropout of 50% was added to each fully connected layer. The concatenation block consists of 3 channel-wise concatenations connected to 6 convolutional layers. The first channel-wise concatenation is via a 1×1 convolutional layer of 64 kernel size and the second channel-wise concatenation is via two 1×1 convolutional layers of kernel size 64 and 128, respectively. The third channel-wise concatenation is via three 1×1 convolutional layers of kernel size 64, 256, and 256, respectively. The model is trained on 30 epochs with a learning rate of 0.0002, using Adam as the optimizer. To minimize overfitting, we used a 50% dropout for regularization and batch normalization (BN). The rectified linear units (ReLU) non-linearity was used as the activation function for all layers, and the learning rate was controlled using the adaptive moment estimation (Adam) optimizer. The similarity between images was determined using the absolute distance, after which the values were passed through a sigmoid activation function to yield a similarity score, and the loss function was defined by computing the contrastive loss, as shown in Equation (2).

(2) $$L_{\left(w_{1}x_{1}x_{2}\right)}=\frac{1}{2}\sum_{x=1}\;\left[y\ln a+\left(1-y\right)ln\;\left(1-a\right)\right]$$

where *y* is the label, *x* is the input, and α is the predicted outcome. Here, $x_{1}$ and $x_{2}$ are a pair of CXR images passed into the separate identical CNNs. *w* is the shared parameter vector that neural networks will learn; $f(x_{1})$ and $f(x_{2})$ are the latent representation vectors of the input. If $X_{1}$, $X_{2}$ are same, then the $||\;f\left(x_{1}\right)-f\left(x_{2}\right)\;{\left|\right|}^{2}$ is small, meaning that they are similar with the same label, and if $x_{1}$, $x_{2}$ are different, then the $||\;f\left(x_{1}\right)-f\left(x_{2}\right)\;{\left|\right|}^{2}$ is large, which means that they are not similar. Therefore, the absolute distance between the paired images is given in Equation (3).

(3) $$d\left(x_{1},x_{2}\right)=||f\left(x_{1}\right)-f\left(x_{2}\right){\left|\right|}^{2}$$

### 3.6. Wavelet

Wavelets are a type of function that can be used to scale and localize a function. The wavelet transform cuts up the input image into different frequency constituents, and then studies each constituent with a resolution suited to its scale. The underlying concept behind the wavelet transform is to extend and convert the input image in the time domain using a wavelet basis, which then decomposes it into a series of sub-band components with different image resolutions, frequency attributes, and directional features. In order to achieve dimensionality reduction, low-frequency constituents are maintained while high-frequency constituents are eliminated as much as possible in the wavelet transform. A wavelet is a ‘tiny wave’ function, generally indicated as ψ(·), defined over the main axis (−∞,∞). It must fulfill three basic properties to be classified as a wavelet, as presented in Equations (4) and (5). The integral of ψ(·) is zero, as presented in Equation (4):

(4) $$\int_{-\infty }^{\infty }\psi \left(u\right)du=0$$

The integral of the square of ψ(·) is unity, as presented in Equation (5):

(5) $$\int_{-\infty }^{\infty }\psi^{2}\left(u\right)du=1$$

Equation (6) explicitly expresses the admissibility condition:

(6) $$C_{\psi }=\int_{0}^{\infty }\frac{{\left|\psi \left(f\right)\right|}^{2}}{f}\;dfsatisfies\;0<C_{\psi }<\infty$$

By converting and stretching this mother wavelet as shown in Equation (7), a two-fold indexed family of wavelets can be formed:

(7) $$\psi_{\lambda ,t}\left(u\right)=\frac{1}{\sqrt{\lambda }}\psi \left(\frac{u-t}{\lambda }\right)$$

where λ>0 and *t* is 1; the normalization on the right-hand side of Equation (7) is chosen such that $\left|\right|\psi_{\lambda ,t}\left|\right|=\left||\psi |\right|$ for all λ, *t* and $\frac{1}{\sqrt{\lambda }}$ is the normalizing term.

### 3.7. Multi-Resolution Analysis (MRA)

The core of wavelet principle is multi-resolution analysis (MRA), which divides an image into wavelets (wave-like functions) that are scaled and time-shifted copies of the genuine or mother wavelet. Low- and high-pass filters are implemented using the scaling and wavelet functions, respectively. As a result, the image is sub-sampled to distinguish low and high frequencies after passing via the low- and high-pass filters. The relationship between the decomposition components and the original image f(t) is expressed in Equation (8).

(8) $$f\left(t\right)=CA_{4}+CD_{4}+CD_{3}+CD_{2}+CD_{1}$$

where f(t) is the original image; $CA_{4}$ is the approximate component of the fourth-level decomposition; $CD_{4}$, $CD_{3}$, $CD_{2}$, and $CD_{1}$ are the detail components for the fourth-, third-, second-, and first-level decompositions, respectively. DWT is a method used by MRA to describe a time-varying signal in respect to frequency constituents. The genuine image is disintegrated into many other images with varying levels of resolution (scale). The image f(t) is disintegrated into scaling and wavelet functions, which can be expressed mathematically as in Equation (9).

(9) $$f\left(t\right)=\sum_{k}A_{j}\left(k\right)⌀\left(t-k\right)+\sum_{k}\sum_{j=0}^{j=1}D_{j}\left(k\right)2^{\left(j⁄2\right)}\psi \left(2^{j}t-k\right)$$

**Figure 4:**
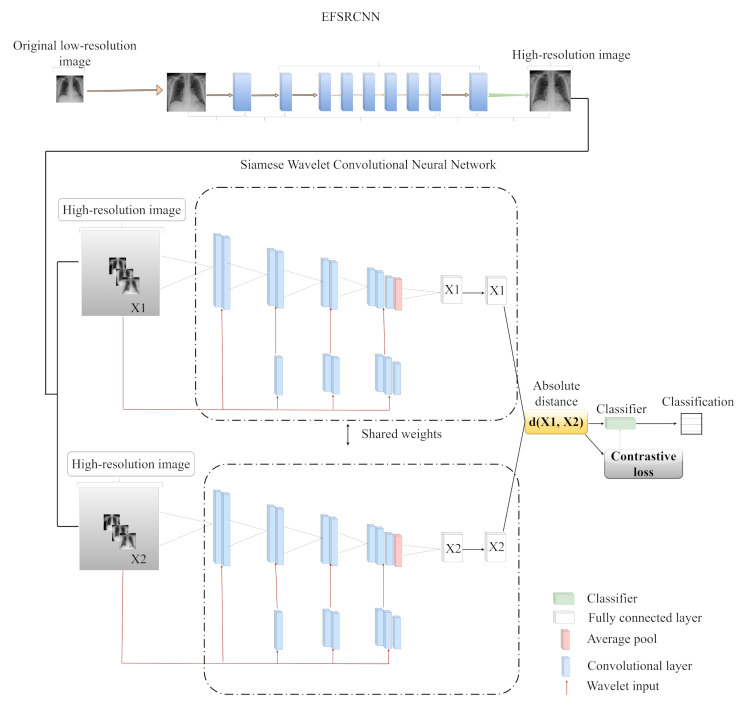
The proposed super-resolution-based Siamese wavelet multi-resolution convolutional neural network for COVID-19 classification (COVID-SRWCNN).

As shown in Figure 4, the wavelet function $\psi (2^{j}t-k)$ generates the low-frequency constituents (detailed) of the disintegrated image, while the scaling function ⌀(t−k) generates the high-frequency constituents (approximate). These frequency constituents were derived using a filter bank with low-pass and high-pass filters for detail and approximate, respectively. The wavelet is scaled by a factor of two for every stage of decomposition. The high-frequency constituent is broken down again to obtain more information about the input image. The beginning section of the right-hand side is a projection of f(t) in the scaling space, with coefficients $A_{j}\left(k\right)$ representing image f(t)’s discrete smoothing approximations, and the other section is a projection of f(t) in the wavelet space, with coefficients $D_{j}\left(k\right)$ representing image f(t)’s discrete informative features of the image that are the wavelet transform coefficients. Wavelet multi-resolution analysis is widely used and efficient in image processing applications. Centered on an improved wavelet multi-resolution analysis CNN, this paper uses this technique to create a Siamese wavelet multi-resolution convolutional neural network for COVID-19 classification tasks, as illustrated in Figure 4. For the image input, the discrete wavelet transform (DWT) is presented numerically in Equations (10) and (11).

(10) $$W_{⌀}\left(j_{0},z_{1},z_{2}\right)=\frac{1}{\sqrt{M\times N}}\sum_{x=1}^{M}\sum_{y=1}^{N}I(x,y){⌀}_{j_{0},z_{1},z_{2}}\;x,y$$

(11) $$W_{\psi }\left(j_{0},z_{1},z_{2}\right)=\frac{1}{\sqrt{M\times N}}\sum_{x=1}^{M}\sum_{y=1}^{N}I(x,y)\psi_{j_{0},z_{1},z_{2}}^{i}\;x,y$$

where M×N indicates the image dimension, I(x,y) is the pixel intensity at position (x,y), ⌀ and ψ are the scaling and wavelet functions, and i=(H,V,D) is the wavelet function’s path index. The wavelet function generates 4 sub-bands for one image at separate levels ψ: smooth version (LL), vertical borders (LH), horizontal borders (HL), and diagonal borders (HH) of the image.

### 3.8. The Proposed Super-Resolution Wavelet Multi-Resolution CNN (COVID-SRWCNN)

Our proposed COVID-SRWCNN is an integrated super-resolution CNN and Siamese wavelet convolutional neural network for diagnosing COVID-19 from chest X-rays, as presented in Figure 4. The proposed architecture consists of the super-resolution part, which handles the image enhancement by reconstructing high-resolution images from low-resolution image counterparts as the first part, while the second part is the Siamese wavelet multi-resolution convolutional neural network, which extracts and learns high-dimensional feature vectors from the super-resolution imagery generated by the super-resolution network for COVID-19 classification. We adopted some evaluation metrics, such as the receiver operating characteristic (ROC), area under curve (AUC), accuracy (ACC), sensitivity (SEN), and specificity (SPE).

## 4. Results

### 4.1. Experimental Setup

We collected a public dataset of chest X-ray photographs from three open sources to evaluate the performance of our proposed algorithm in screening COVID-19. To further verify the efficacy of our proposed model, we carried out two stages of experiments, where the first stage considered the complete proposed model and the second stage considered the proposed model without the super-resolution section, as presented in Table 3. For a fair comparison, we ran 11 famous ImageNet pre-trained models and four state-of-the-art COVID-19 methods on the same dataset, as presented in Table 4 and Table 5. From all indications, our proposed model outperforms the other methods and deep learning models, with promising performance.

### 4.2. Implementation Details

In this study, the dataset is divided into three portions, and each class label has the same number of CXR images. The training, validation, and test partitions contain 60%, 20%, and 20%, respectively. In this work, the whole training approach can be viewed in two stages:

(1) The enhanced fast super-resolution convolutional neural network reconstructs high-resolution images from the original scaled low-resolution images.

(2) The SWMRCNN network is constructed using super-resolution (SR) imagery. The reconstructed high-resolution image is fed as input to the SWMRCNN framework for COVID-19 classification. We trained the overall end-to-end network (COVID-SRWCNN), which consists of EFSRCNN + SWMRCNN, on the NVIDIA GTX1070. Keras was used for the construction of the proposed COVID-SRWCNN scheme. To construct our batch, we paired a single image with two separate images. If the images were the same, we labeled the pair as one; otherwise, we labeled it zero. This pairing process was repeated for a total of 10,800 images and thus amounted to 21,600, from which 16,800 belong to the training pairs and 4800 belong to the validation pairs. This is one of the significant advantages of the Siamese neural network. We can generate a large number of training pairs using a relatively smaller number of training images. In this work, we adopted a CNN as the base network. We introduce a Softmax with 12 units in the last fully connected layer. However, the remaining 1200 images were tested by pairing them with the training images.

## 5. Evaluation

The evaluation is divided into two sections, the first of which demonstrates the benefits of the super-resolution network in the image reconstruction process in terms of PSNR and SSIM. In the second section of the report, Equations (12)–(15) are used to evaluate the classification network. The following assessment criteria were used to assess the performance of our proposed method: accuracy (ACC), precision (PRE), sensitivity (SEN), specificity (SPE), and area under curve (AUC).

(12) $$F_{1}=2\times \frac{Precision\times Recall}{Precision+Recall}$$

(13) $$Accuracy=\frac{TP+TN}{TP+TN+FP+FN}$$

(14) $$Sensitivity=\frac{TP}{TP+FN}$$

(15) $$Specificity=\frac{TN}{TN+FP}$$

where TP, FP, and FN indicate the outcomes of true positive, false positive, and false negative, respectively.

### 5.1. Super-Resolution Evaluation

**Figure 5:**
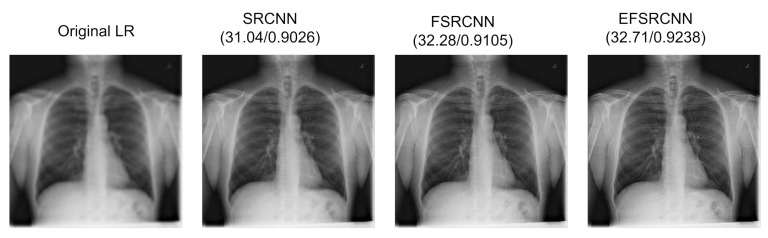
Comparison of the quantitative results of our proposed EFSRCNN with other selected state-of-the-art models using the same dataset. The PSNR value is reported on the left while the SSIM value is reported on the right for the whole region.

**Figure 6:**
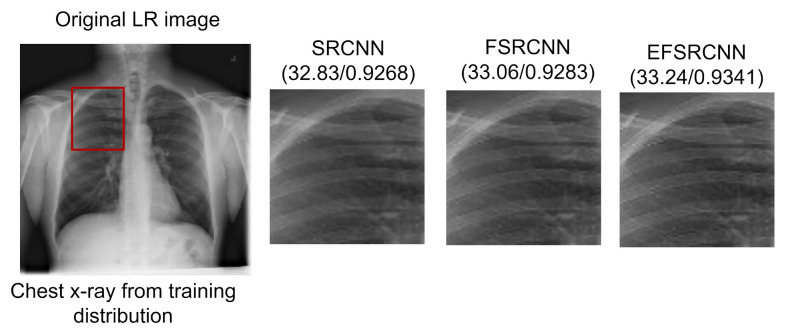
Comparison of the quantitative results of our proposed EFSRCNN with other selected state-of-the-art models using the same dataset. The PSNR value is reported on the left while the SSIM value is reported on the right for the region of interest.

We demonstrate the performance of our proposed super-resolution model by comparing it with well-known state-of-the-art models, namely SRCNN and FSRCNN. The comparison of these methods is based on the implementation of their source code, using the same dataset for fairness. The main focus of the study is the PSNR and the test time, in which our proposed EFSRCNN proves to be the fastest. Our proposed method still outweighs previous methods on PSNR and SSIM values. From the experiment, EFSRCNN achieves satisfactory performance in run time and restoration quality by redesigning the FSRCNN structure. Table 6 shows the structural configuration of SRCNN, FSRCNN, and our proposed EFSRCNN. Table 7 summarizes the quantitative results of our proposed model, while Figure 5 and Figure 6 provide visual examples in comparison with other state-of-the-art models.

### 5.2. COVID-19 Classification Evaluation

**Figure 7:**
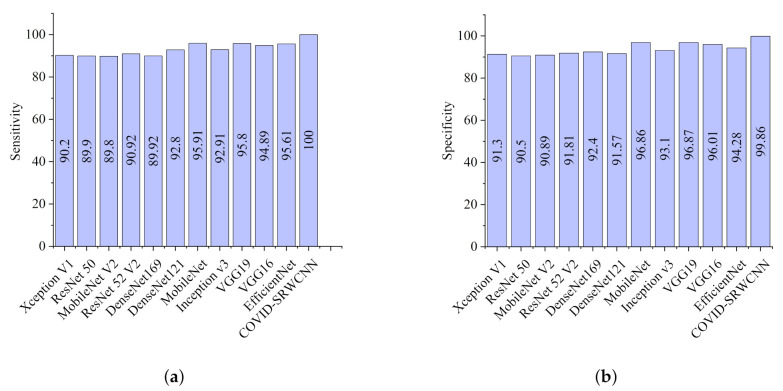
Performance report of our model and selected pre-trained models. (**a**) Sensitivity report for the selected deep pre-trained models and our proposed model. (**b**) Specificity report for the selected deep pre-trained models and our proposed model.

**Figure 8:**
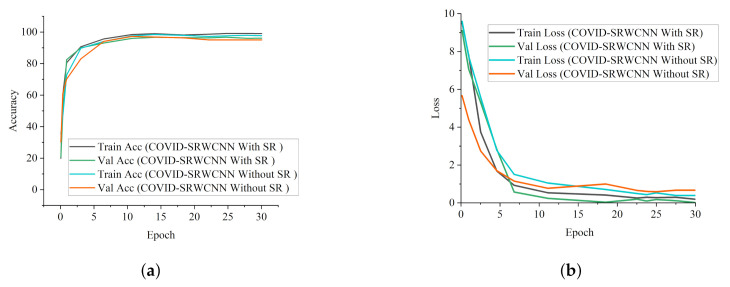
Training and validation report of our model with and without super resolution (SR). (**a**) Accuracy curves showing the performance of our proposed COVID-SRWCNN with and without super resolution (SR). (**b**) Loss curves reported for our proposed COVID-SRWCNN with and without super resolution (SR).

**Figure 9:**
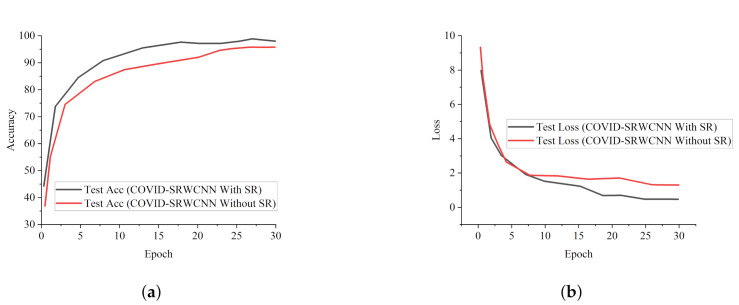
Test report of our model with and without super resolution (SR). (**a**) Test accuracy curves showing the performance of our proposed COVID-SRWCNN with and without super resolution (SR). (**b**) Test loss curves reported for our proposed COVID-SRWCNN with and without super resolution (SR).

**Figure 10:**
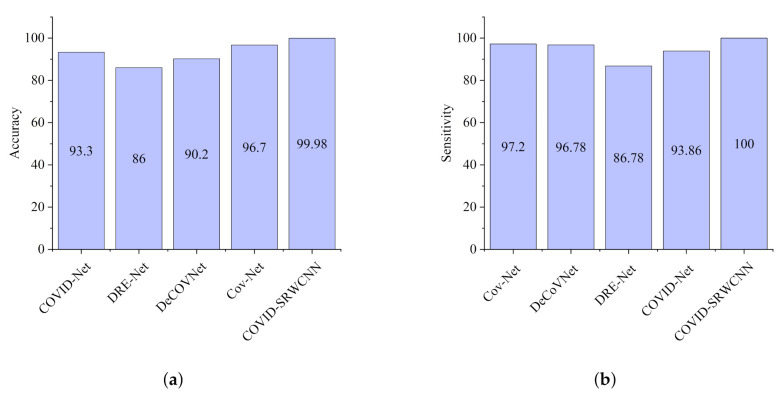
Comparison report for the selected state-of-the-art COVID-19 models and our proposed model. (**a**) Accuracy report for the selected state-of-the-art COVID-19 models and our proposed model. (**b**) Sensitivity report for the selected state-of-the-art COVID-19 models and our proposed model.

**Figure 11:**
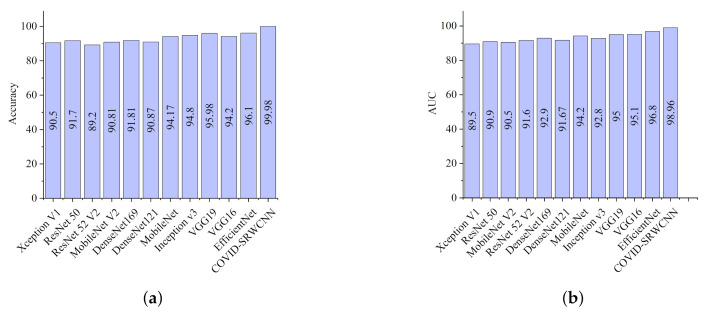
Comparison report for the selected deep pre-trained models and our proposed model. (**a**) Accuracy report for the selected deep pre-trained models and our proposed model. (**b**) AUC report for the selected deep pre-trained models and our proposed model.

**Figure 12:**
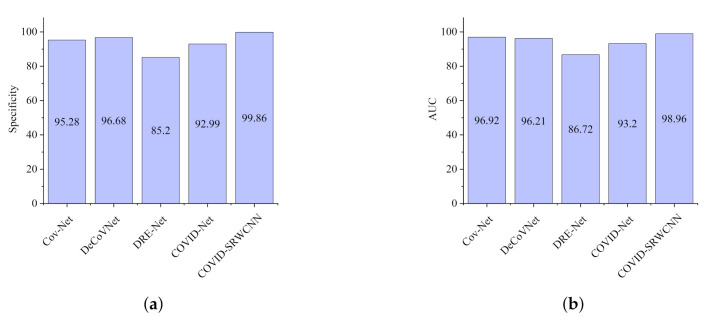
Comparison report for the selected state-of-the-art COVID-19 models and our proposed model. (**a**) Specificity report for the selected state-of-the-art COVID-19 models and our proposed model. (**b**) AUC report for the selected state-of-the-art COVID-19 models and our proposed model.

The experimental results show that our proposed COVID-SRWCNN architecture outweighs state-of-the-art COVID-19 models and some selected deep learning models pre-trained on ImageNet. For fairness, all implementations are based on their source code using the same CXR dataset. From the experimental analysis of our comparative report, as presented in Figure 7a, MobileNet V2 achieves the lowest sensitivity score of 89.8%, whereas ResNet50 obtains the lowest specificity score of 90.5%, as depicted in Figure 7b. From all indications, our proposed model outweighs all the pre-trained models, with a high sensitivity score of 99.78% and a 98.86% specificity score. We conducted an ablation study to evaluate the contribution and effect of the super-resolution technique on the performance of the proposed framework. The first model is termed COVID-19-SRWCNN with SR while the second model is termed COVID-19-SRWCNN without SR, as depicted in Table 3. Figure 8a shows the training and validation accuracy of both models with smooth progression and steady convergence. The training and validation loss of both models, showing a gradual reduction in loss, are presented in Figure 8b. Moreover, the test accuracy and loss are presented in Figure 9a,b, respectively. From all indications, the proposed model with super resolution (SR) shows satisfactory performance in training, validation, and test accuracy with commendable loss reduction. Moreover, the accuracy and sensitivity performance of the selected state-of-the-art COVID-19 models are reported in comparison with our proposed model in Figure 10a,b. Our model performs better than the state-of-the-art models, achieving high accuracy of 98.98% and 100% sensitivity, followed by Cov-Net, with 96.75% accuracy and 97.2% sensitivity. We also compared our proposed model with selected pre-trained models in terms of accuracy and AUC, as presented in Figure 11a,b. Specificity and area under curve (AUC) are other important performance metrics that we adopted in comparison with the state-of-the-art COVID-19 methods, as presented in Figure 12a,b. Among the selected state-of-the-art COVID-19 models and pre-trained models, Cov-Net and EfficientNet show good performance; however, our proposed model achieves the best AUC value of 98.96%. In the course of our work, we reviewed several studies related to COVID-19 diagnosis based on artificial intelligence and presented some comparisons. Some studies reported few performance indicators to support their claims, as seen in Table 8. More importantly, our proposed model achieves better performance, with more indicators reported compared to the other state-of-the-art COVID-19 methods cited from the literature. For fair comparison, we compared our proposed model with selected state-of-the-art COVID-19 models using the same dataset, as presented in Table 5. DeepPneumonia [49] obtained the lowest accuracy score of 90.06%, followed by COVID-Net [56] with 93.32%.

**Figure 13:**
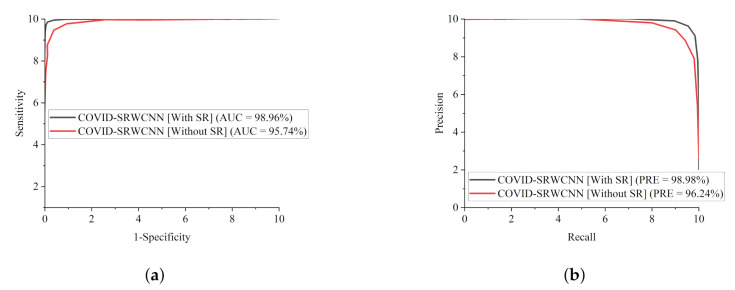
Comparison report of our proposed COVID-SRWCNN with and without super resolution (SR). (**a**) ROC–AUC curves of our proposed COVID-SRWCNN with and without super resolution (SR). (**b**) Precision–recall curves of our proposed COVID-SRWCNN with and without super resolution (SR).

**Figure 14:**
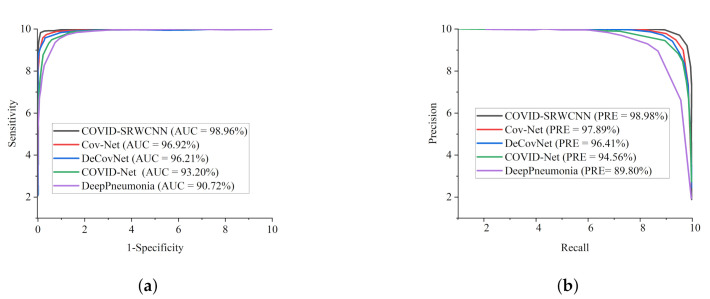
Comparison report of our proposed COVID-SRWCNN in comparison with selected state-of-the-art COVID-19 models using the same dataset. (**a**) ROC–AUC curves of our proposed COVID-SRWCNN in comparison with selected state-of-the-art COVID-19 models using the same dataset. (**b**) Precision–recall curves of our proposed COVID-SRWCNN in comparison with selected state-of-the-art COVID-19 models using the same dataset.

To further validate the efficacy of our proposed COVID-SRWCNN model, We adopted ROC and precision–recall metrics. For diagnosing sensitive conditions such as COVID-19, it is important to adopt ROC as a method to measure the overall accuracy, as well as the precision–recall curve to measure the mean average precision of our model. Figure 13a shows the ROC curves for the two-stage experiment conducted with super resolution (SR) and without super resolution (SR), while the precision–recall curve is presented in Figure 13b. We went a step further to compare our proposed model with some selected state-of-the-art COVID-19 methods in terms of ROC and precision–recall, as presented in Figure 14a,b.

Finally, it is worth mentioning that all the models were trained on the same dataset for fair comparison. We only modified the last layer of the models to correspond to the number of class labels in our dataset. From all indications, our proposed COVID-SRWCNN outperformed the other models in terms of precision–recall and ROC. The precision–recall graphs show that the curves of our proposed model are the closest to the upper-right corner of the graph with the largest area, and therefore has higher precision associated with higher sensitivity. Similarly, the ROC graphs indicate that the curves of our proposed model are the closest to the upper-left corner of the graph with the largest area under the curve, and therefore has higher sensitivity associated with higher specificity. More importantly, as mentioned above, the stated result in terms of receiver operating characteristic (ROC) and precision–recall can assist expert radiologists in striking a balance between accuracy and precision.

#### Comparative Study

We compare the findings of our proposed model with previous up-to-date COVID-19 screening methods. To diagnose COVID-19 from CT and CXR scans, a number of studies have been performed. We compare the results of the proposed wavelet-integrated CNN model to previously published research. U-Net was used by Chen et al. [38] to extract high-resolution features from CT. COVID-19 is detected using a CNN approach by Wang et al. [48], who achieved 93.3% accuracy, 87.6% sensitivity, and 95.5% specificity. Our model obtained much higher results than Wang et al. [48] in terms of accuracy, sensitivity, and specificity, with a margin of 5.89% , 12.19%, and 4.36%, respectively. COVID-19 is classified by Shi et al. [44] using a random forest technique, which achieved 87.9% accuracy, 83.3% sensitivity, and 90.7% specificity. To discover COVID-19, Jin et al. [45] used a logistic regression approach. Li et al. [42] suggested a ResNet50 model for classifying COVID-19 with a method of weight sharing. To detect COVID-19, Jin et al. [46] built an AI-based approach. Xu et al. [47] and Wang et al. [56] present remarkable research, although only a few indicators are mentioned. To detect COVID-19 from CT images, Song et al. [49] used a deep learning algorithm. Zhang et al. [37] proposed an 18-layer residual CNN pre-trained on ImageNet with a separate anomaly detection mechanism for the classification of COVID-19. The authors recorded an impressive result of 90.7% sensitivity and 90.7% specificity, whereas our model achieved much higher results in comparison with Zhang et al. [37], as depicted in Table 4, with a margin of 8.30% and 8.16% in sensitivity and specificity, respectively. The results of the aforementioned procedures are summarized in Table 8. Mohamed et al. [57] proposed a COVID-19 algorithm using hybridization and swarm-based models for image classification. Using MobileNetV3 for the feature extraction and Aquila as the optimizer, the proposed framework was tested on two datasets of both CXR and CT COVID-19 scans. The comparison results show the high performance of the proposed model over other methods. Dalia et al. [58] presented four different fractional-order cuckoo search optimization algorithms (FO-CS) using heavy-tailed distributions from COVID-19 datasets. The FO-CS model introduced in the classification task achieved high accuracy performance when compared to other approaches. Mucahid et al. [59] proposed the detection of COVID-19 using machine learning algorithms by introducing different patch sizes of the CT images. An SVM classifier and different cross-validation value were applied for the classification task. Thus, the best performance for accuracy was 99.68% using Grey-Level Size Zone Matrix (GLSZM) feature extraction methods and 10-fold cross-validation. As demonstrated in Table 8, our suggested model has competitive efficiency for COVID-19 diagnosis. In comparison to famous deeper neural networks and the selected COVID-19 state-of-the-art models, as presented in Table 4 and Table 5, our model is capable of handling small-scale datasets with significantly lower computing costs, as presented in Table 5.

According to [60], the manual detection of COVID-19 by an expert utilizing CXR can have high sensitivity but low specificity of 25%. This inadequate specificity leads to false-positive predictions, which leads to ineffective therapy and a waste of money. Our suggested model, COVID-SRWCNN, has high specificity of 98.86%, and it can be used to help expert radiologists to reduce the number of false-positive instances reported. More importantly, the stated result in terms of the receiver operating characteristic (ROC) can aid expert radiologists in achieving a balance of accuracy and precision.

Furthermore, some comments on COVID-SRWCNN’s computational cost and model complexity are necessary. We avoided the use of max pooling at each convolutional block by using the wavelet transform, which reduced the model complexity and computation time. Another intriguing feature of our COVID-SRWCNN is its capacity to minimize noise in input images by concatenating the combination of the generated detail coefficients at each decomposition level to each convolutional block through a 1×1 convolutional layer. In terms of computing costs, our model was trained on an NVIDIA GTX 1080. For the implementation of our architecture, we used the Keras framework.

## 6. Discussion

For the performance enhancement of our proposed network (COVID-SRWCNN), we integrated distinct input images into the convolutional neural networks via channel-wise concatenation. The sole purpose of introducing wavelet multi-resolution analysis (WMRA) is to provide a varying depiction of the input images at different scales to achieve full-spectral analysis. DWT can interpret the input images at various scales. While it is general knowledge that CNNs process images mainly in the spatial domain and only partially on the spectral domain, WMRA allows for the full-spectral processing of images, resulting in these algorithms possessing different properties.

By the integration of WMRA into the convolutional neural network, it enhances the network’s ability to obtain the magnitude of the frequency data that are not found both in the average pooling and the convolutional layers, which comprehensively reduces the spectral analysis. In addition to this, at different phases, wavelets extract the required multi-resolution spectral information from the input data. In a manner that is similar to the process of pooling, a multi-resolution analysis of the data used as input will show the input in various scales. Wavelet transform works in such a way that every sub-sampling stage can be seen as a distinct pooling process. This caused us not to employ wavelet transform as a clear substitute for the pooling layers in the proposed framework utilized in this study; instead, we incorporated wavelet transform to extract information from the input data and pass it into the convolutional layers.

If the output of the estimated wavelet transform for every image is added to the convolutional neural network with the intention of adding the wavelet coefficients generated at different decomposition levels, it will amount to the loss of multi-scale information. This act will limit or restrict the proposed network from learning insightful details from the CXR data at varying resolutions. One of the aspects that we have deemed necessary in solving this dilemma is multi-scale input processing, where the CXR images are used for analysis at various resolutions in every stage of wavelet decomposition. To accomplish not only different resolution analysis but also low- and high-frequency domain analysis, various decomposition phases of wavelet transform are integrated into the CNN.

## 7. Conclusions

In this work, we proposed a CNN-based super-resolution with a Siamese wavelet multi-resolution framework for COVID-19 classification, with the aim of addressing the challenge of the low-resolution characteristics of CXR images. We utilized our proposed enhanced fast super-resolution CNN to solve the problem of CXR’s low quality by reconstructing high-resolution images from their low-resolution counterparts. Finally, our modified Siamese wavelet multi-resolution CNN was used to extract meaningful features from the reconstructed high-resolution CXR images for the classification of COVID-19. We have shown that our model has the ability to reconstruct high-resolution images that are similar to the ground-truth low-resolution images and further captures deep features for the classification of COVID-19. By a well-observed margin, our proposed COVID-SRWCNN performs better than some famous pre-trained models and some previously proposed state-of-the-art COVID-19 diagnosis techniques.

## Figures and Tables

**Table 1:** Description of the chest X-ray dataset showing different categories of pneumonia illnesses and the distribution of images per category as well as the number of selected images per category.

S/N	Pneumonia	Data Count	Selected No	Train Set	Val Set	Test Set
1	Atelectasis	4999	1000	700	200	100
2	Bacteria	3029	1000	700	200	100
3	Cardiomegaly	10,000	1000	700	200	100
4	Consolidation	10,000	1000	700	200	100
5	COVID-19	3616	1000	700	200	100
6	Effusion	10,000	1000	700	200	100
7	Infiltration	10,000	1000	700	200	100
8	Mass	10,000	1000	700	200	100
9	Nodule	10,000	1000	700	200	100
10	Pneumothorax	10,000	1000	700	200	100
11	Healthy	10,000	1000	700	200	100
12	Viral	2983	1000	700	200	100
Total		94,627	12,000	8400	2400	1200

**Table 2:** Transitional configuration from SRCNN and FSRCNN to EFSRCNN.

	SRCNN	FSRCNN	Transistion State	EFSRCNN
First layer	Conv (9, 64, 1)	Conv (5, 56, 1)	Conv (3, 56, 1)	Conv (3, 48, 1)
Middle layer	Conv (5, 32, 64)	Conv (1, 12, 56) 4 Conv (3, 12, 12) Conv (1, 56, 12)	Conv (1, 12, 56) 5 Conv (3, 12, 12) Conv (1, 56, 12)	Conv (1, 12, 48) 6 Conv (3, 12, 12) Conv (1, 48, 12)
Last layer	Conv (5, 1, 32)	DeConv (9, 1, 56)	DeConv (9, 1, 56)	DeConv (9, 1, 48)
Input size	HR	LR	HR	HR
Model size	57,184	12,464	8653	5178
Speed	8.7×	41.3×	46.8×	52.1×

**Table 3:** We conducted two-stage experiments for the purpose of evaluating the influence of the SR network on the classification performance of COVID-SRWCNN. The first experiment considered COVID-SRWCNN with the SR network and the second experiment considered COVID-SRWCNN without the SR network. The result shows that our proposed enhanced fast SR network is effective in achieving high restoration quality.

Model	ACC (%)	AUC (%)	SEN (%)	SPE (%)	PRE (%)	Time (min)
COVID-SRWCNN (With SR)	98.98	98.96	99.78	98.86	98.98	21.8
COVID-SRWCNN (Without SR)	94.68	95.74	97.85	95.84	96.24	17.2

**Table 4:** Performance comparison of selected deep pre-trained models. From all indications, our proposed COVID-SRWCNN exhibits the highest score with the best performance.

Models	PRE (%)	SEN (%)	SPE (%)	ACC (%)	AUC (%)
Xception V1	85.60	90.20	91.30	90.50	89.50
ResNet 50	86.70	89.90	90.50	91.71	90.90
MobileNet V2	87.70	89.82	90.89	89.23	90.51
ResNet 52 V2	89.60	90.92	91.81	90.81	91.64
DenseNet169	90.20	89.92	92.43	91.81	92.92
DenseNet121	92.65	92.83	91.57	90.87	91.67
MobileNet	95.67	95.91	96.86	94.17	94.23
Inception V3	93.78	92.91	93.13	94.83	92.81
VGG19	94.86	95.81	96.87	95.98	95.03
VGG16	95.96	94.89	96.01	94.25	95.12
EfficientNet	96.78	95.61	94.28	96.15	96.83
COVID-SRWCNN	98.98	99.78	98.86	98.98	98.96

**Table 5:** Comparison of our proposed COVID-SRWCNN model with other selected state-of-the-art COVID-19 models using the same training data distribution.

COVID-19 Models	AUC (%)	SPE (%)	SEN (%)	ACC (%)	PRE (%)	Time (min)
COVID-Net [56]	93.20	92.99	93.86	93.32	94.56	23.9
DeCoVNet [51]	96.21	96.68	96.78	96.21	96.41	24.7
Cov-Net [42]	96.92	95.28	97.20	96.75	97.89	26.2
COVID-SRWCNN	98.96	98.86	99.78	98.86	98.98	21.8

**Table 6:** We compare the structural configuration of the SRCNN, FSRCNN, and our proposed EFSRCNN, including their reported PSNR using the same chest X-ray dataset.

	SRCNN	FSRCNN	EFSRCNN
First layer	Conv (9, 64, 1)	Conv (5, 56, 1)	Conv (3, 48, 1)
Middle layer	Conv (5,32, 64)	Conv (1, 12, 56) 4 Conv (3, 12, 12) Conv (1, 56, 12)	Conv (1, 12, 48) 6 Conv (3, 12, 12) Conv (1, 48, 12)
Last layer	Conv (5, 1, 32)	DeConv (9, 1, 56)	DeConv (9, 1, 48)
Input size	HR	LR	HR
Model size	57,184	12,464	5178
Speed	12.7×	48.3×	52.1×

**Table 7:** We compare the PSNR and SSIM results of the SRCNN, FSRCNN, and our proposed EFSRCNN using the same chest X-ray dataset.

	Metrics	SRCNN	FSRCNN	EFSRCNN
Region of Interest	PSNR SSIM	32.83 dB 0.9268	33.06 dB 0.9283	32.24 dB 0.9341
Whole region	PSNR SSIM	31.04 dB 0.9026	32.28 dB 0.9105	32.71 dB 0.9238

**Table 8:** We compare our proposed COVID-SRWCNN model with state-of-the-art COVID-19 image-based diagnosis models.

Literature	Architecture	Performance (%)
Wang et al. [56]	2D CNN	82.9 (ACC)
Shi et al. [44]	Random forest-based CNN	87.9 (ACC) 83.3 (SEN) 90.7 (SPE)
Chen et al. [38]	2D Unet ++	95.2 (ACC) 100.0 (SEN) 93.6 (SPE)
Li et al. [42]	2D ResNet 50	90.0 (SEN) 96.0 (SPE)
Song et al. [49]	2D ResNet 50	86.0 (ACC)
Jin et al. [45]	2D Unet++ and 2D CNN	97.4 (SEN) 92.2 (SPE)
Xu et al. [47]	2D CNN	86.7 (ACC)
Jin et al. [46]	2D CNN	94.1 (SEN) 95.5 (SPE)
Wang et al. [48]	3D ResNet and attention	93.3 (ACC) 87.6 (SEN) 95.5 (SPE)
Zhang et al. [37]	2D Unet and 2D CNN	90.7 (SEN) 90.7 (SPE)
COVID-SRWCNN	Super-Resolution CNN and Wavelet	99.79 (ACC) 99.78 (SEN) 99.86 (SPE) 98.96 (AUC) 98.98 (PRE)

## Data Availability

In this study, we collected chest X-ray data of different pneumonia-related illnesses from three different open sources. We collected 3616 scans of COVID-19 CXR from the COVID-19 radiography database. We collected 3029 scans of bacterial pneumonia, 8851 scans of healthy patients, and 2983 scans of viral pneumonia from the Kaggle database of the Radiological Society of North America (RSNA). Moreover, we collected 74,999 scans of other pneumonia-related illnesses from the National Institute of Health (NIH). Link 1: Available online: https://www.kaggle.com/tawsifurrahman/covid19-radiography-database (accessed on 12 May 2021). Link 2: Available online: https://www.kaggle.com/c/rsna-pneumonia-detection-challenge/data (accessed on 12 May 2021). Link 3: Available online: https://www.kaggle.com/nih-chest-xrays/data (accessed on 12 May 2021).

## References

[1] COVID-19 Dashboard by the Center for Systems Science and Engineering (CSSE) at Johns Hopkins University (JHU). https://coronavirus.jhu.edu/map.html.

[2] World Health Organization Coronavirus (COVID-19) Dashboard. https://covid19.who.int/.

[3] Wang W., Xu Y., Gao R., Lu R., Han K., Wu G., Tan W. (2020). Detection of SARS-CoV-2 in different types of clinical specimens. JAMA Am. Med. Assoc..

[4] Jeong E.K., Park O., Park Y.J., Park S.Y., Kim Y.M., Kim J., Jo J., Kim J., Kim T., Kweon S. (2020). Coronavirus disease-19: The first 7,755 cases in the Republic of Korea. Osong Public Health Res. Perspect..

[5] Chen C., Gao G., Xu Y., Pu L., Wang Q., Wang L., Wang W., Song Y., Chen M., Wang L. (2020). SARS-CoV-2–positive sputum and feces after conversion of pharyngeal samples in patients with COVID-19. Ann. Intern. Med. Am. Coll. Physician.

[6] Ai T., Yang Z., Hou H., Zhan C., Chen C., Lv W., Tao Q., Sun Z., Xia L. (2020). Correlation of Chest CT and RT-PCR Testing for Coronavirus Disease 2019 (COVID-19) in China: A Report of 1014 Cases. Radiology.

[7] Hosseiny M., Kooraki S., Gholamrezanezhad A., Reddy S., Myers L. (2020). Radiology Perspective of Coronavirus Disease 2019 (COVID-19): Lessons From Severe Acute Respiratory Syndrome and Middle East Respiratory Syndrome. Am. J. Roentgenol..

[8] Ulhaq A., Khan A., Gomes D., Pau M. (2020). Computer vision for covid-19 control: A survey. arXiv.

[9] Nneji G.U., Cai J., Deng J., Monday H.N., James E.C., Lemessa B.D., Yutra A.Z., Leta Y.B., Nahar S. COVID-19 Identification Using Deep Capsule Network: A Perspective of Super-Resolution CNN on Low-Quality CXR Images. Proceedings of the 2021 the 7th International Conference on Communication and Information Processing (ICCIP 2021).

[10] Nneji G.U., Cai J., Deng J., Monday H.N., James E.C., Ukwuoma C.C. (2022). Multi-Channel Based Image Processing Scheme for Pneumonia Identification. Diagnostics.

[11] Monday H.N., Li J.P., Nneji G.U., James E.C., Chikwendu I.A., Ejiyi C.J., Oluwasanmi A., Mgbejime G.T. The capability of multi resolution analysis: A case study of COVID-19 diagnosis. Proceedings of the 2021 the 4th International Conference on Pattern Recognition and Artificial Intelligence (PRAI 2021).

[12] Nneji G.U., Cai J., Jianhua D., Monday H.N., Chikwendu I.A., Oluwasanmi A., James E.C., Mgbejime G.T. Enhancing low quality in radiograph datasets using wavelet transform convolutional neural network and generative adversarial network for COVID-19 identification. Proceedings of the 2021 the 4th International Conference on Pattern Recognition and Artificial Intelligence (PRAI 2021).

[13] Monday H.N., Li J.P., Nneji G.U., Oluwasanmi A., Mgbejime G.T., Ejiyi C.J., Chikwendu I.A., James E.C. Improved convolutional neural multi-resolution wavelet network for COVID-19 pneumonia classification. Proceedings of the 2021 the 4th International Conference on Pattern Recognition and Artificial Intelligence (PRAI 2021).

[14] Monday H.N., Li J., Nneji G.U., Nahar S., Hossin M.A., Jackson J. (2020). COVID-19 Pneumonia Classification Based on NeuroWavelet Capsule Network. Healthcare.

[15] Nneji G.U., Deng J., Monday H.N., Hossin M.A., Obiora S., Nahar S., Cai J. (2022). COVID-19 Identification from Low-Quality Computed Tomography Using a Modified Enhanced Super-Resolution Generative Adversarial Network Plus and Siamese Capsule Network. Healthcare.

[16] Wong H.Y.F., Lam H.Y.S., Fong A.H.-T., Leung S.T., Chin T.W.-Y., Lo C.S.Y., Lui M.M.-S., Lee J.C.Y., Chiu K.W.-H., Chung T.W.-H. (2020). Frequency and distribution of chest radiographic findings in patients positive for COVID-19. Radiol. Radiol. Soc. N. Am..

[17] Ravishankar H., Sudhakar P., Venkataramani R., Thiruvenkadam S., Annangi P., Babu N., Vaidya V. (2016). Understanding the mechanisms of deep transfer learning for medical images. Deep Learning and Data Labeling for Medical Applications.

[18] Yu Y., Lin H., Meng J., Wei X., Guo H., Zhao Z. (2017). Deep transfer learning for modality classification of medical images. Information.

[19] Ko H., Chung H., Kang W.S., Park C., Kim D.W., Kim S.E., Chung C.R., Ko R.E., Lee H., Seo J.H. (2020). An Artificial Intelligence Model to Predict the Mortality of COVID-19 Patients at Hospital Admission Time Using Routine Blood Samples: Development and Validation of an Ensemble Model. J. Med. Internet Res..

[20] dos Santos Santana Í.V., da Silveira A.C.M., Sobrinho Á., e Silva L.C., da Silva L.D., Santos D.F.S., Gurjão E.C., Perkusich A. (2021). Classification Models for COVID-19 Test Prioritization in Brazil: Machine Learning Approach. J. Med. Internet Res..

[21] Chen Y., Ouyang L., Bao F.S., Li Q., Han L., Zhang H., Zhu B., Ge Y., Robinson P., Xu M. (2021). A Multimodality Machine Learning Approach to Differentiate Severe and Nonsevere COVID-19: Model Development and Validation. J. Med. Internet Res..

[22] Liu T., Tsang W., Xie Y., Tian K., Huang F., Chen Y., Lau O., Feng G., Du J., Chu B. (2021). Preferences for Artificial Intelligence Clinicians Before and During the COVID-19 Pandemic: Discrete Choice Experiment and Propensity Score Matching Study. J. Med. Internet Res..

[23] Xu M., Ouyang L., Han L., Sun K., Yu T., Li Q., Tian H., Safarnejad L., Zhang H., Gao Y. (2021). Accurately Differentiating Between Patients With COVID-19, Patients With Other Viral Infections, and Healthy Individuals: Multimodal Late Fusion Learning Approach. J. Med. Internet Res..

[24] Plante T.B., Blau A.M., Berg A.N., Weinberg A.S., Jun I.C., Tapson V.F., Kanigan T.S., Adib A.B. (2020). Development and External Validation of a Machine Learning Tool to Rule Out COVID-19 Among Adults in the Emergency Department Using Routine Blood Tests: A Large, Multicenter, Real-World Study. J. Med. Internet Res..

[25] Ko H., Chung H., Kang W.S., Kim K.W., Shin Y., Kang S.J., Lee J.H., Kim Y.J., Kim N.Y., Jung H. (2020). COVID-19 Pneumonia Diagnosis Using a Simple 2D Deep Learning Framework With a Single Chest CT Image: Model Development and Validation. J. Med. Internet Res..

[26] Montazeri M., ZahediNasab R., Farahani A., Mohseni H., Ghasemian F. (2021). Machine Learning Models for Image-Based Diagnosis and Prognosis of COVID-19: Systematic Review. JMIR Med. Inform..

[27] Yu C.-S., Chang S.-S., Chang T.-H., Wu J.L., Lin Y.-J., Chien H.-F., Chen R.-J. (2021). A COVID-19 Pandemic Artificial Intelligence–Based System with Deep Learning Forecasting and Automatic Statistical Data Acquisition: Development and Implementation Study. J. Med. Internet Res..

[28] Albahli S., Yar G.N.A.H. (2021). Fast and Accurate Detection of COVID-19 Along With 14 Other Chest Pathologies Using a Multi-Level Classification: Algorithm Development and Validation Study. J. Med. Internet Res..

[29] COVID-19 Dataset. https://github.com/ieee8023/covid-chestxray-dataset.

[30] He K., Zhang X., Ren S., Sun J. (2016). Identity Mappings in Deep Residual Networks. European Conference on Computer Vision.

[31] Ghoshal B., Tucker A. (2020). Estimating Uncertainty and Interpretability in Deep Learning for Coronavirus (COVID-19) Detection. arXiv.

[32] Narin A., Kaya C., Pamuk Z. (2021). Automatic detection of coronavirus disease (COVID-19) using X-ray images and deep convolutional neural networks. Pattern Anal. Appl..

[33] Szegedy C., Vanhoucke V., Ioffe S., Shlens J., Wojna Z. Rethinking the Inception Architecture for Computer Vision. Proceedings of the IEEE Conference on Computer Vision and Pattern Recognition (CVPR).

[34] He K., Zhang X., Ren S., Sun J. Deep residual learning for image recognition. Proceedings of the 2016 IEEE Conference on Computer Vision and Pattern Recognition (CVPR).

[35] Szegedy C., Ioffe S., Vanhoucke V., Alemi A. (2016). Inception-v4, inception-resnet and the impact of residual connections on learning. arXiv.

[36] Russakovsky O., Deng J., Su H., Krause J., Satheesh S., Ma S., Huang Z., Karpathy A., Khosla A., Bernstein M. (2015). Imagenet large scale visual recognition challenge. Int. J. Comput. Vis..

[37] Zhang J., Xie Y., Li Y., Shen C., Xia Y. (2020). Covid-19 screening on chest X-ray images using deep learning based anomaly detection. arXiv.

[38] Chen J., Wu L., Zhang J., Zhang L., Gong D., Zhao Y., Chen Q., Huang S., Yang M., Yang X. (2020). Deep learning-based model for detecting 2019 novel coronavirus pneumonia on high-resolution computed tomography. Sci. Rep..

[39] Zhou Z., Rahman Siddiquee M.M., Tajbakhsh N., Liang J. (2018). Unet++: A nested u-net architecture for medical image segmentation. Deep Learning in Medical Image Analysis and Multimodal Learning for Clinical Decision Support.

[40] Shan F., Gao Y., Wang J., Shi W., Shi N., Han M., Xue Z., Shen D., Shi Y. (2020). Lung infection quantification of COVID-19 in CT images with deep learning. arXiv.

[41] Gozes O., Frid-Adar M., Greenspan H., Browning P.D., Zhang H., Ji W., Bernheim A., Siegel E. (2020). Rapid ai development cycle for the coronavirus (Covid-19) pandemic: Initial results for automated detection & patient monitoring using deep learning ct image analysis. arXiv.

[42] Li L., Qin L., Xu Z., Yin Y., Wang X., Kong B., Bai J., Lu Y., Fang Z., Song Q. (2020). Artificial intelligence distinguishes COVID-19 from community acquired pneumonia on chest CT. Radiology.

[43] Nneji G.U., Cai J., Monday H.N., Hossin M.A., Nahar S., Mgbejime G.T., Deng J. (2022). Fine-Tuned Siamese Network with Modified Enhanced Super-Resolution GAN Plus Based on Low-Quality Chest X-ray Images for COVID-19 Identification. Diagnostics.

[44] Shi F., Xia L., Shan F., Wu D., Wei Y., Yuan H., Jiang H., Gao Y., Sui H., Shen D. (2020). Large-Scale Screening of COVID-19 from Community Acquired Pneumonia Using Infection Size-Aware Classification. arXiv.

[45] Jin S., Wang B., Xu H., Luo C., Wei L., Zhao W., Hou X., Ma W., Xu Z., Zheng Z. (2020). AI-assisted CT imaging analysis for COVID-19 screening: Building and deploying a medical AI system in four weeks. Appl. Soft Comput..

[46] Jin C., Chen W., Cao Y., Xu Z., Zhang X., Deng L., Zheng C., Zhou J., Shi H., Feng J. (2020). Development and evaluation of an AI system for COVID-19 diagnosis. medRxiv.

[47] Xu X., Jiang X., Ma C., Du P., Li X., Lv S., Yu L., Chen Y., Su J., Lang G. (2020). Deep Learning System to Screen novel Coronavirus Disease 2019 Pneumonia. Engineering.

[48] Wang S., Kang B., Ma J., Zeng X., Xiao M., Guo J., Cai M., Yang J., Li Y., Meng X. (2020). A deep learning algorithm using CT images to screen for Corona Virus Disease (COVID-19). medRxiv.

[49] Song Y., Zheng S., Li L., Zhang X., Zhang X., Huang Z., Chen J., Wang R., Zhao H., Zha Y. (2021). Deep learning enables accurate diagnosis of novel coronavirus (COVID-19) with CT images. IEEE/ACM Trans. Comput. Biol. Bioinform..

[50] Tang Z., Zhao W., Xie X., Zhong Z., Shi F., Liu J., Shen D. (2020). Severity assessment of coronavirus disease 2019 (COVID-19) using quantitative features from chest CT images. arXiv.

[51] Zheng C., Deng X., Fu Q., Zhou Q., Feng J., Ma H., Liu W., Wang X. (2020). Deep learning-based detection for COVID-19 from chest CT using weak label. IEEE Trans. Med. Imaging.

[52] Wolff R.F., Moons K.G.M., Riley R.D., Whiting P.F., Westwood M., Collins G.S., Reitsma J.B., Kleijnen J., Mallett S. (2019). PROBAST: A tool to assess the risk of bias and applicability of prediction model studies. Ann. Intern. Med..

[53] Rahman T., Khandakar A., Qiblawey Y., Tahir A., Kiranyaz S., Kashem S.B.A., Islam M.T., Al Maadeed S., Zughaier S.M., Khan M.S. (2021). Exploring the effect of image enhancement techniques on COVID-19 detection using chest X-ray images. Comput. Biol. Med..

[54] (2020). Radiological Society of North America (RSNA). https://www.kaggle.com/c/rsna-pneumonia-detection-challenge/data.

[55] NIH Chest X-rays|Kaggle [Internet]. Https://www.kaggle.com/nih-chest-xrays/data.

[56] Wang L., Lin Z.Q., Wong A. (2020). COVID-Net: A tailored deep convolutional neural network design for detection of COVID-19 cases from chest X-ray images. Sci. Rep..

[57] Elaziz M.A., Dahou A., Alsaleh N.A., Elsheikh A.H., Saba A.I., Ahmadein M. (2021). Boosting COVID-19 Image Classification Using MobileNetV3 and Aquila Optimizer Algorithm. Entropy.

[58] Yousri D., Elaziz M.A., Abualigah L., Oliva D., Al-Qaness M.A., Ewees A.A. (2020). COVID-19 X-ray images classification based on enhanced fractional-order cuckoo search optimizer using heavy-tailed distributions. Appl. Soft Comput..

[59] Barstugan M., Ozkaya U., Öztürk Ş. (2021). Coronavirus (COVID-19) Classification Using CT Images by Machine Learning Methods.

[60] Xu B., Xing Y., Peng J., Zheng Z., Tang W., Sun Y., Xu C., Peng F. (2020). Chest CT for detecting COVID-19: A systematic review and meta-analysis of diagnostic accuracy. Eur. Radiol..

